# Dry Eye Symptoms and Ocular Pain in Veterans with Glaucoma

**DOI:** 10.3390/jcm8071076

**Published:** 2019-07-22

**Authors:** Aubrey R Tirpack, Elizabeth Vanner, James M Parrish, Anat Galor, Hong-Uyen Hua, Sarah R Wellik

**Affiliations:** 1 Bascom Palmer Eye Institute, University of Miami Miller School of Medicine, 900 NW 17th Street, Miami, FL 33136, USA; 2 Bruce W. Carter VA Medical Center, 1201 N.W. 16th St., Miami, FL 33125, USA; 3 Roski Eye Institute, University of Southern California School of Medicine, 1450 San Pablo St., Los Angeles, CA 90033, USA

**Keywords:** dry eye, dry eye disease, glaucoma, glaucoma medications, ocular pain

## Abstract

Dry eye and glaucoma are two frequently encountered ocular conditions, which can lead to substantial morbidity and decreased quality of life. Patients on topical glaucoma medications are known to be at greater risk for ocular surface symptoms. Veterans seen in the eye clinics at the Miami Veterans Affairs Hospital from January to July 2016 completed surveys assessing dry eye and ocular pain symptoms, including the five item Dry Eye Questionnaire (DEQ5). A total of 62 patients with glaucoma completed the survey. Of those, 52 were on glaucoma medications at the time of the survey, with the majority requiring more than one medication to control intraocular pressure. The frequency of mild or greater dry eye symptoms (defined as DEQ5 >6) tended to increase with increasing medication burden, and patients on brimonidine were more likely to report a DEQ5 >6. Patients on three or more glaucoma medications were more likely to report symptoms of shooting pain, dryness, and itchiness. Patients using timolol were more likely to report throbbing and pain by light, while those on latanoprost reported stinging. Our data support an association between increasing number of glaucoma medications and worsening of dry eye symptoms. Patient and medication-associated symptoms can be used to tailor individual medication regimens.

## 1. Introduction

Glaucoma is an optic neuropathy defined by optic disc cupping, characteristic visual field defects, and often an increase in intraocular pressure. It remains a leading cause of irreversible blindness, with an estimated 60.5 million people affected worldwide [1,2]. The burden of this visually devastating disease is only expected to increase amid the rapid growth of the aging population, with an estimated 112 million people affected by the year 2040 [3]. The treatment of glaucoma consists of a step-wise approach to lower intraocular pressure, typically beginning with pressure lowering eye drops and/or laser trabeculoplasty, with surgical intervention traditionally reserved for more advanced disease. While glaucoma medications are useful to lower intraocular pressure and slow progression of glaucomatous damage, they carry associated side effects, including ocular surface irritation and discomfort. Numerous studies have shown a high prevalence of dry eye symptoms in patients with glaucoma, specifically in those treated with glaucoma medications [4,5,6,7,8,9].

Dry eye often presents with symptoms of discomfort and blurred vision. It is seen more commonly in the elderly population, with an estimated prevalence of approximately 19% in those over the age of 75 [10]. As the frequency of glaucoma also increases with age, it is expected that many patients carry both diagnoses. Given the morbidity and decreased quality of life associated with dry eye, numerous surveys have been designed to accurately diagnosis and quantify patients’ symptoms. The five item Dry Eye Questionnaire (DEQ5) and the Ocular Surface Disease Index (OSDI) are two such validated surveys [11,12]. Additional questionnaires to assess ocular pain symptoms such as the Neuropathic Pain Symptom Inventory modified for the eye (NPSI-Eye) and the short form McGill Pain Questionnaire (sf-MPQ) are used in clinical practice [13]. These surveys provide physicians with personalized clinical information, which can be used to target specific therapies and address patients’ individual needs.

Prior studies at the Miami Veterans Affairs Hospital have shown a high prevalence of dry eye in the veteran population [14]. In these patients, ocular surface symptoms were associated with the use of glaucoma medications, and severity of dry eye symptoms was correlated with reduced quality of life [15,16]. There is a lack of published literature specifically addressing ocular pain in those patients with glaucoma. In this paper, we thus focused specifically on dry eye and ocular pain symptoms in a glaucoma population, as this information can guide physicians in future treatment decisions.

## 2. Experimental Section

This is a single-center, cross-sectional study of patients evaluated at the Miami Veterans Affairs Hospital between January 2016 and July 2016. Patients seen in the ophthalmology and optometry clinics over the designated time period were invited to complete standardized eye questionnaires, including the DEQ5, OSDI, NPSI-Eye, and sf-MPQ, to assess their dry eye and ocular pain symptoms. These surveys were modified for the eye and are validated forms designed to evaluate dry eye and ocular pain symptoms. The DEQ5 questionnaire assesses the frequency and intensity of dry eye symptoms, with scores ranging from 0 (no symptoms) to 22 (severe symptoms), and a score of >6 indicating mild or greater dry eye symptoms. The OSDI survey is a 12 item questionnaire assessing dry eye symptoms (including grittiness, soreness, and pain by light) and the effect on vision. The sf-MPQ survey evaluates the severity of 15 pain and affective descriptors (throbbing, shooting, stabbing, sharp, cramping, gnawing, hot-burning, aching, heavy, tender, splitting, tiring-exhausting, sickening, fearful, and punishing-cruel). Additional pain descriptors found in other DE questionnaires were added to the sf-MPQ survey, including dryness, itchiness, irritating, piercing, and stinging. The NPSI-Eye is a validated 10 item tool to assess neuropathic pain (including the symptom of pins and needles) [17]. The questionnaires were self-administered by the patients, with assistance provided when needed.

After administration of the surveys, the medical records of patients completing the questionnaires were retrospectively reviewed for baseline characteristics and demographics including age, sex, diagnosis of glaucoma, number and type of glaucoma medications used, and a history of glaucoma surgery or laser. The only combination glaucoma medication used at this institution was dorzolamide/timolol, which was recorded as the two separate components for the purposes of this study. Systemic comorbidities were also examined, including a history of post-traumatic stress disorder, depression, headaches, migraines, back pain, fibromyalgia, sleep apnea, diabetes, and hypertension. Exclusion criteria included current contact lens use, active infection, presence of pterygium or corneal edema, history of refractive surgery, eyelid pathology (such as ectropion, lagophthalmos), and cataract surgery within the preceding 6 months. Systemic exclusion criteria included a diagnosis of Sjögrens syndrome, human immunodeficiency virus, sarcoidosis, graft-versus-host disease, or collagen vascular disease.

Statistical analysis was performed using Microsoft^®^ Excel Version 16.16 and SAS version 9.4 (Cary, NC, USA). Independent samples *t*-test, Mann–Whitney U, Jonckheere-Terpstra, chi square, Fisher’s exact tests, Spearman and Kendall correlations, and logistic regression were used as appropriate to compare variables of interest. A *p*-value < 0.05 was considered statistically significant. The project was started as a quality assurance study. The Bruce W. Carter VA Medical Center later approved the retrospective review of patient questionnaires and charts. The research adhered to the tenets of the Declaration of Helsinki and complied with the Health Insurance Portability and Accountability Act of 1996.

## 3. Results

A total of 151 patients were seen in the ophthalmology and optometry clinics at the Miami Veterans Affairs Hospital and completed the dry eye and ocular pain questionnaires over the designated time period. Sixty-two of these patients (41.1%) had a known diagnosis of glaucoma at the time of survey administration and met the inclusion criteria (Table 1). The majority of glaucoma patients were male (*n* = 58, 93.5%) and the average age was 72 years old (range 46–96, SD = 10.5).

Fifty-two patients were on glaucoma medications at the time of the survey administration (83.9%). The most commonly used medication was timolol (*n* = 40), followed by latanoprost (*n* = 39), dorzolamide (*n* = 36), and brimonidine (*n* = 22). The majority of patients required more than one topical glaucoma medication to control intraocular pressure: 1 medication (*n* = 9), 2 medications (*n* = 11), 3 medications (*n* = 22), and 4 medications (*n* = 10). Of the 22 patients on brimonidine, none were on brimonidine alone, and all were on three or more glaucoma medications. Of the 40 patients on timolol, only one (2.5%) was on timolol alone, and of the 39 patients on latanoprost, eight (21%) were on latanoprost alone. A total of 18 patients had a history of prior incisional glaucoma surgery, with eight patients having undergone trabeculectomy and 13 patients having had tube shunt surgery. Eleven patients had a history of laser trabeculoplasty.

**Figure 1:**
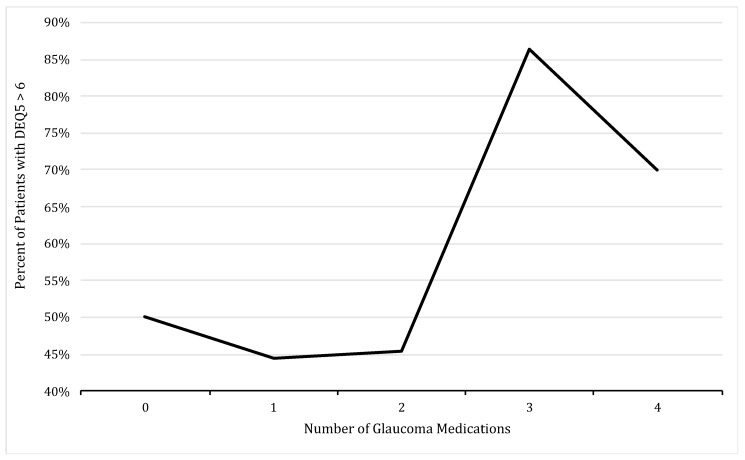
Percent of glaucoma patients, classified by number of medications used, with a five item Dry Eye Questionnaire (DEQ5) score of >6, indicating mild or greater dry eye symptoms.

All patients completed the DEQ5 questionnaire. Patients on three or more glaucoma medications at the time of survey administration were more likely to have a DEQ5 >6, indicating mild or greater dry eye symptoms: 50.0% on 0 medications, 44.4% on one medication, 45.5% on two medications, 86.4% on three medications, and 70.0% on four medications (*p* = 0.004) (Figure 1). In total, 81.3% of patients on three or more glaucoma medications reported a DEQ5 >6, compared to only 46.7% of patients on fewer than three medications (*p* = 0.004). Additionally, those patients on three or more glaucoma medications tended to report more severe dry symptoms, with an average DEQ5 score of 8.5 in patients on no medications, 7.4 in patients on 1–2 medications, and 9.9 in patients on 3–4 medications. Overall, individuals with a DEQ5 ≤6 (*n* = 16) were on an average of 1.7 medications, while those reporting a DEQ5 >6 (*n* = 46) were on an average of 2.5 medications (*p* = 0.03).

Patients on brimonidine were more likely than those not on brimonidine to report mild or greater dry eye symptoms, as defined by a DEQ5 >6 (81.8% vs. 55.0%, *p* = 0.03). There was a trend for those on brimonidine to more frequently report a DEQ5 >6 compared to those on other glaucoma eye medications: 81.8% on brimonidine, 72.5% on timolol, 69.4% on dorzolamide, 69.2% on latanoprost. When considering both each individual medication and number of medications in a forward stepwise multivariable analysis, only brimonidine remained significantly associated with mild or greater dry eye symptoms (OR = 3.68, 95% CI 1.06–12.85, *p* = 0.04).

Ocular pain surveys were reviewed to analyze symptom-specific complaints. Specifically, the results of the sf-MPQ, OSDI, and NPSI-Eye survey were used to evaluate pain and effective symptoms. The most common descriptors reported by surveyed glaucoma patients included itchiness (*n* = 35), irritating (*n* = 29), dryness (*n* = 28), and aching (*n* = 21). Patients on three or more glaucoma medications were more likely than patients on fewer medications to report symptoms of shooting pain (28% vs. 4%, *p* = 0.03), dryness (67% vs. 38%, *p* = 0.04), and itchiness (74% vs. 44%, *p* = 0.02). Medication-specific ocular pain symptoms were also investigated (Table 2). Those patients on timolol were more likely than those not on timolol to report symptoms of throbbing (41.2% vs. 13.3%, *p* = 0.03) and pain by light (42.4% vs. 11.1%, *p* = 0.02). Patients using latanoprost were more likely than those not using latanoprost to report stinging (37.5% vs. 10.5%, *p* = 0.04).

The association between glaucoma medication use and the presence of certain systemic comorbidities (headache, migraine, back pain, fibromyalgia, diabetes, post-traumatic stress disorder, depression, sleep apnea, and hypertension) was investigated, and those comorbidities reaching statistical significance are presented in Table 2. Patients using brimonidine were more likely to report a history of headaches compared to those not on brimonidine (15.0% vs. 0.0%, *p* = 0.04). There was a moderately significant association between depression and latanoprost use, compared to those not on latanoprost (41.0% vs. 17.4%, *p* = 0.055). There was no association between specific glaucoma medication use and the concomitant use of antidepressant medication and anti-anxiety medications.

For each specific ocular pain and systemic comorbidity found to be statistically associated with one of the four glaucoma medications used in our series (Table 2), a univariate logistic regression model was built for that symptom and medication to determine an odds ratio (Table 3). Additionally, bivariate logistic regression models were built for that symptom and medication, with each of the other three medications or the total numbers of other medications as a second explanatory variable to further explore these associations. It is of note that only three patients in our series reported a history of headaches, and therefore no bivariate analysis could be attempted. The results of these analyses are in Table 3. In all these bivariate models, the second explanatory variable was not statistically significant. However, in the three models with brimonidine as the second explanatory variable, there were reduced odds of the specific dry eye symptom associated with using brimonidine, especially pain by light and stinging, for which there were clinically significant decreased odds of approximately one half.

## 4. Discussion

Glaucoma and ocular surface disease are two frequently encountered ocular conditions, which account for a substantial number of office visits and can lead to patient morbidity. While many studies have shown a correlation between the use of glaucoma medications and the incidence of dry eye disease, none have evaluated specific ocular pain descriptors to better quantify and understand medication-associated symptoms. The use of validated tests in this study (DEQ5, OSDI, NPSI-Eye, sf-MPQ) allowed for both the evaluation of dry eye symptoms and pain-specific descriptors.

The majority of patients in our series required the use of topical pressure lowering eye medications for treatment of their glaucoma. The most commonly used eye medication was timolol, followed closely by latanoprost and dorzolamide. Fewer than half of patients were using brimonidine at the time of survey administration. These prescription patterns mirror other epidemiological studies, which have shown topical beta blockers and prostaglandin analogs to be the most commonly used glaucoma medications [18,19,20]. The majority of our patients required more than one glaucoma medication to control their intraocular pressure, with 69.4% using two or more medications at the time of survey administration. This is an important observation, as polypharmacy alone has been shown to affect patient adherence, with increasing missed doses and time between refills seen in patients with more complicated dosing regimens [21,22,23,24].

The DEQ5 survey was administered to all patients to better assess the frequency and intensity of their dry eye symptoms. Those patients on three or more glaucoma medications were more likely to report the presence of mild or greater dry eye symptoms (measured by a DEQ5 >6) and more severe symptoms (measured as a higher DEQ5 score) than those patients on a fewer number of mediations. This corroborates prior studies, which have shown a higher frequency of dry eye symptoms in patients on glaucoma medications, with increased incidence and severity amongst those using multiple medications [4,8,16]. All patients in our series were using preserved glaucoma medications, which contain benzalkonium chloride (BAK). Such preservatives have been shown to negatively affect the lipid layer of the tear film and decrease the density of goblet cells, leading to reduced stability of the tear film [4,25,26]. Patients treated with multiple BAK-containing glaucoma medications have increased clinical evidence of dry eye (as measured by lissamine green staining) compared to those patients using fewer BAK-containing medications [4]. BAK concentrations also vary between glaucoma medications (latanoprost: BAK 0.02%, timolol: BAK 0.01%, dorzolamide: BAK 0.0075%, brimonidine: BAK 0.005%, and combination dorzolamide/timolol: BAK 0.0075%), though a clear dose and concentration dependent effect on corneal toxicity has not been firmly established [27].

Interestingly, patients using brimonidine at the time of survey administration were statistically more likely than those not on brimonidine to report a DEQ5 >6. Brimonidine, an alpha-2-adrenergic receptor agonist, is known to have a number of ocular side effects, including conjunctival hyperemia, allergic conjunctivitis, and ocular pruritis, which limits its use in some patients [28]. As previously discussed, brimonidine is not a typical ‘first-line’ glaucoma medication (in contrast to more frequently prescribed prostaglandin analogs and beta-blockers), and is traditionally added later in the treatment course as additive therapy for uncontrolled intraocular pressure or in those patients intolerant to other glaucoma medications. This is consistent with our findings that of the 22 patients on brimonidine, none were on brimonidine alone and all were on three or more glaucoma medications. As our study found both brimonidine and the use of three or more glaucoma medications increased the likelihood of mild or greater dry eye (DEQ5 >6), further analysis was required to determine if this was truly a symptom specific to brimonidine use, or simply a reflection of polypharmacy and the use of multiple preservative-containing medications. 

Unique to our study was the use of standardized surveys to investigate patient-specific descriptors of ocular pain. Patients with glaucoma in our series most commonly described symptoms of itching, irritation, dryness, and aching. Those patients on three or more glaucoma medications were statistically more likely to report symptoms of shooting pain, dryness, and itching compared to those on fewer topical medications. Interestingly, patients using timolol were more likely to report symptoms of throbbing and pain by light compared to those not on timolol. Timolol, a medication which has been used for decades in the treatment of glaucoma, is a non-selective beta-adrenergic blocker which lowers intraocular pressure by decreasing the production of aqueous humor in the eye. Known side effects include central nervous system changes (depression, fatigue, memory loss), cardiovascular effects (bradycardia and hypotension), respiratory complications (bronchospasm), and ocular side effects, including deep orbital pain and burning [29,30]. Though further investigation is needed to clarify these ocular pain descriptors, the symptoms of throbbing and pain by light noted in patients using timolol in our study may be similar to these established medication side effects, further supporting a medication-associated finding. Those patients on latanoprost were more likely to report stinging than those patients not using this medication. Latanoprost, a prostaglandin analog, is often used as first line therapy for treatment of glaucoma, given its excellent intraocular pressure lowering properties and limited systemic side effect profile. It does have well-documented ocular side effects, including conjunctival hyperemia, elongation of eyelashes, iris darkening, and periocular skin pigmentation [31]. Interestingly, while stinging is a known side effect of prostaglandin analog use, prior studies have found it to be less frequently reported in patients using latanoprost compared to those using timolol or fixed combination dorzolamide–timolol [32,33]. Dorzolamide, a carbonic anhydrase inhibitor, which is associated with side effects of stinging, burning, and a bitter taste, was not found to be statistically associated with any dry eye or ocular pain symptoms in our study. While future studies with a greater number of patients are needed to confirm the medication-specific pain descriptors seen in our series, such information can be vital in helping providers target patient complaints and determine the need to switch or discontinue a certain glaucoma medication.

Interestingly, further investigation of these medication-associated pain descriptors in a bivariate model, which included brimonidine as the second explanatory variable, showed that brimonidine had an odds ratio of less than 1.00 for the descriptors analyzed (suggesting that use of brimonidine is associated with reduced odds of experiencing this ocular pain symptom). This was particularly true for pain by light and stinging, for which there were clinically significant decreased odds of approximately one-half in those patients on brimonidine. Therefore, while patients using brimonidine were at increased odds of dry eye symptoms (DEQ 5>6), they were less likely to report these specific pain descriptors. It is plausible that this may represent a case of reverse causality, with physicians being less likely to prescribe brimonidine to those patients with specific ocular pain symptoms.

The relationship between multiple systemic comorbidities and glaucoma medication use was evaluated. Patients in our series on latanoprost tended to have a more frequent diagnosis of depression, which was of borderline significance. Traditionally, topical beta-blockers (timolol) have been associated with symptoms of depression, memory loss, and disorientation [28]. While it is not clear from the current literature why prostaglandin analog use would be associated with depression, this is a finding which should be explored in future studies. Additionally, those patients on brimonidine were more likely to report a history of headache. Headache, fatigue, and drowsiness are known systemic side effects of topical alpha-agonist use, however prior studies have failed to show a difference in headaches between patients on brimonidine or timolol [28,34]. Our study is limited by the specific patient population investigated, with veteran patients tending to be older and predominantly male. As such, our results may not be generalizable to a broader glaucoma patient population. In addition, we did not have information on all potential confounders, such as glaucoma duration. Additionally, our study lacked a control population of patients without glaucoma, which would provide more information on the relative risk of pain symptoms in our patient population. Given the time period of survey administration, newer glaucoma medications, including Rho-kinase inhibitors, were not yet available. Future studies will be needed to assess prescription patterns, medication tolerability, and medication-associated symptoms for these newer medications. Additionally, the surveys administered to patients in this study quantified ocular pain without specification of laterality. While many patients used glaucoma medications in both eyes, some used specific medications in only one eye. Additionally, a potentially confounding factor is that 29% of patients in our cohort had a history of incisional surgery in one or both eyes. Future studies will need to utilize these validated questionnaires to assess eye-specific, as opposed to patient-specific symptoms.

## 5. Conclusions

Patients on three or more glaucoma medications are more likely to have dry eye symptoms compared to those patients on fewer medications. They are also more likely to report specific ocular surface symptoms of dryness, shooting pain, and itchiness. This is the first study to use validated ocular pain surveys to assess patient-specific symptoms. The use of brimonidine was shown to be associated with a higher frequency of dry eye symptoms and a more frequent history of headaches. Patients on timolol were more likely to report throbbing and pain by light, while those on latanoprost reported more stinging. Dorzolamide was not associated with any specific increase in ocular pain descriptors. Given the known association between glaucoma medications and ocular surface disease, providers should actively assess dry eye symptoms in their glaucoma patients, note patient-specific pain descriptors, and tailor a medication regimen based on a patient’s individual complaints. More research is needed to understand the associations between glaucoma medications and dry eye symptoms, including how dry eye symptoms and medication intolerability may accelerate the transition from medically treated to surgically treated glaucoma.

## Figures and Tables

**Table 1:** Baseline glaucoma patient characteristics.

Number of Patients	62
Age [mean (+/−SD)]	72 years (+/−10.5)
Sex	
Male [*n* (%)]	58 (93.5)
Female [*n* (%)]	4 (6.5)
Current glaucoma medication use [*n* (%)]	52 (83.9)
Timolol [*n* (%)]	40 (64.5)
Latanoprost [*n* (%)]	39 (62.9)
Dorzolamide [*n* (%)]	36 (58.1)
Brimonidine [*n* (%)]	22 (35.5)
Number of glaucoma medications [*n* (%)]	
No medications [*n* (%)]	10 (16.1)
One medication [*n* (%)]	9 (14.5)
Two medications [*n* (%)]	11 (17.7)
Three medications [*n* (%)]	22 (35.5)
Four medications [*n* (%)]	10 (16.1)
Prior laser trabeculoplasty [*n* (%)]	11 (17.7)
Prior trabeculectomy [*n* (%)]	8 (12.9)
Prior tube shunt [*n* (%)]	13 (21.0)

Abbreviations: SD = standard deviation, *n* = number.

**Table 2:** Relationship between glaucoma medication use and dry eye/ocular pain symptoms and medical comorbidities.

Symptom/Comorbidity	Glaucoma Medication	Patients Using Medication with Symptom [Proportion (Percent)]	Patients Not Using Medication with Symptom [Proportion (Percent)]	*p*-Value ^1^
DEQ5 >6	Latanoprost	27/39 (69.2%)	13/23 (56.5%)	0.31
	Timolol	32/40 (80.0%)	14/22 (63.6%)	0.08 ^†^
	Dorzolamide	25/36 (69.4%)	15/26 (57.7%)	0.34
	Brimonidine	20/22 (90.9%)	26/40 (65.0%)	0.03 *
Throbbing	Latanoprost	13/32 (40.6%)	3/19 (15.8%)	0.06 ^†^
	Timolol	14/34 (41.2%)	2/17 (11.8%)	0.03 *
	Dorzolamide	11/30 (36.7%)	5/21 (23.8%)	0.33
	Brimonidine	6/18 (33.3%)	10/33 (30.3%)	0.82
Pain by light	Latanoprost	10/31 (32.3%)	6/20 (30.0%)	0.87
	Timolol	14/33 (42.4%)	2/18 (11.1%)	0.02 *
	Dorzolamide	12/31 (38.7%)	4/20 (20.0%)	0.16
	Brimonidine	6/20 (30.0%)	10/31 (32.3%)	0.87
Stinging	Latanoprost	12/32 (37.5%)	2/19 (10.5%)	0.04 *
	Timolol	8/33 (24.2%)	6/18 (33.3%)	0.53 ^#^
	Dorzolamide	8/31 (25.8%)	6/20 (30.0%)	0.74
	Brimonidine	4/18 (22.2%)	10/33 (30.3%)	0.74 ^#^
Headache	Latanoprost	3/36 (8.3%)	0/19 (0.0%)	0.54 ^#^
	Timolol	2/37 (5.4%)	1/18 (5.6%)	1.00 ^#^
	Dorzolamide	3/32 (9.4%)	0/23 (0.0%)	0.27 ^#^
	Brimonidine	3/20 (15.0%)	0/35 (0.0%)	0.04 ^#,^*
Depression	Latanoprost	16/39 (41.0%)	4/23 (17.4%)	0.05 ^†^
	Timolol	14/40 (35.0%)	6/22 (27.3%)	0.53
	Dorzolamide	13/36 (36.1%)	7/26 (26.9%)	0.45
	Brimonidine	5/22 (22.7%)	15/40 (37.5%)	0.23

* *p* < 0.05, ^†^ *p* < 0.15 (marginally significant). ^1^ All *p*-values represent Chi-square probabilities unless denoted by ^#^, which indicates Fisher’s exact test; Abbreviations: DEQ5 = Dry Eye Questionnaire 5.

**Table 3:** Odds ratios for univariate and bivariate logistic regression models.

Outcome Variable	Explanatory Variable(s)	Odds	OR 95% CI		*p*-Value	
		Ratio	Low	High		
Analyses for Brimonidine and DEQ5 greater than 6 (first univariate, then bivariates)						
DEQ5 greater than 6	Brimonidine	3.68	1.06	12.85	0.0409	*
DEQ5 greater than 6	Brimonidine	2.98	0.81	11.00	0.1015	^†^
DEQ5 greater than 6	Timolol	1.93	0.61	6.11	0.2619	
DEQ5 greater than 6	Brimonidine	3.80	0.94	15.31	0.0604	^†^
DEQ5 greater than 6	Dorzolamide	0.94	0.28	3.14	0.9199	
DEQ5 greater than 6	Brimonidine	3.59	1.02	12.60	0.0462	*
DEQ5 greater than 6	Latanoprost	1.63	0.54	4.94	0.3862	
DEQ5 greater than 6	Brimonidine	2.92	0.76	11.28	0.1201	^†^
DEQ5 greater than 6	All Other Medications	1.28	0.74	2.22	0.3705	
Analyses for Timolol and Throbbing (first univariate, then bivariates)						
Throbbing	Timolol	5.25	1.03	26.68	0.0456	*
Throbbing	Timolol	6.00	1.10	32.80	0.0386	*
Throbbing	Brimonidine	0.69	0.18	2.60	0.5823	
Throbbing	Timolol	6.32	0.94	42.61	0.0583	^†^
Throbbing	Dorzolamide	0.75	0.16	3.52	0.7105	
Throbbing	Timolol	4.84	0.92	25.35	0.0621	^†^
Throbbing	Latanoprost	3.32	0.77	14.42	0.1090	^†^
Throbbing	Timolol	4.40	0.74	26.32	0.1045	^†^
Throbbing	All Other Medications	1.18	0.57	2.46	0.6574	
Analyses for Timolol and Pain by Light (first univariate, then bivariates)						
Pain by Light	Timolol	5.89	1.16	29.91	0.0323	*
Pain by Light	Timolol	7.65	1.38	42.42	0.0200	*
Pain by Light	Brimonidine	0.49	0.13	1.90	0.3064	
Pain by Light	Timolol	5.67	0.87	36.99	0.0700	^†^
Pain by Light	Dorzolamide	1.07	0.22	5.28	0.9344	
Pain by Light	Timolol	5.90	1.16	30.03	0.0326	*
Pain by Light	Latanoprost	0.99	0.27	3.60	0.9894	
Pain by Light	Timolol	7.43	1.16	47.52	0.0342	*
Pain by Light	All Other Medications	0.81	0.38	1.72	0.5860	
Analyses for Latanoprost and Stinging (first univariate, then bivariates)						
Stinging	Latanoprost	5.10	1.00	26.05	0.0502	^†^
Stinging	Latanoprost	5.72	1.09	30.13	0.0395	*
Stinging	Brimonidine	0.51	0.12	2.07	0.3433	
Stinging	Latanoprost	5.47	1.04	28.61	0.0443	*
Stinging	Dorzolamide	0.65	0.17	2.47	0.5272	
Stinging	Latanoprost	5.68	1.07	30.06	0.0411	*
Stinging	Timolol	0.51	0.13	1.98	0.3292	
Stinging	Latanoprost	6.20	1.12	34.13	0.0362	*
Stinging	All Other Medications	0.71	0.38	1.30	0.2626	
Analyses for Latanoprost and Depression (first univariate, then bivariates)						
Depression	Latanoprost	3.30	0.94	11.57	0.0615	^†^
Depression	Latanoprost	3.65	1.02	13.13	0.0474	*
Depression	Brimonidine	0.42	0.12	1.45	0.1713	
Depression	Latanoprost	3.22	0.92	11.32	0.0683	^†^
Depression	Dorzolamide	1.42	0.46	4.43	0.5426	
Depression	Latanoprost	3.22	0.91	11.34	0.0691	^†^
Depression	Timolol	1.27	0.39	4.13	0.6937	
Depression	Latanoprost	3.35	0.95	11.84	0.0610	^†^
Depression	All Other Medications	0.96	0.59	1.56	0.8740	

* *p* < 0.05, ^†^ *p* < 0.15 (marginally significant). All odds ratios are for a change from no to yes, except for the All Other Medications where the odds ratio is for an increase of one medication.
